# Radiofrequency Ablation for Unstable Ruptured Hepatocellular Carcinoma: A Case Report and Literature Review

**DOI:** 10.7759/cureus.74585

**Published:** 2024-11-27

**Authors:** Matteo Pagani, Carla Tasca, Rosita De Vincenti, Massimo Fedi

**Affiliations:** 1 General Surgery, University of Florence, Florence, ITA; 2 Hepatobiliary Surgery, USL Toscana Centro, Pistoia, ITA; 3 General Surgery, USL Toscana Centro, Pistoia, ITA

**Keywords:** case report, hepatocellular carcinoma rupture, liver bleeding, radiofrequency ablation (rfa), emergency laparotomy

## Abstract

Spontaneous liver bleeding is a rare but life-threatening complication of hepatocellular carcinoma (HCC). The optimal management strategy for this condition remains a topic of ongoing debate. We present the case of a 74-year-old man with cirrhosis and hemorrhagic shock resulting from the spontaneous rupture of HCC. Following a contrast-enhanced CT scan, the patient underwent emergency laparotomy. Hemostasis was attempted using conventional techniques but was unsuccessful. Due to unstable conditions (low blood pressure and high heart rate), poor liver function reserve, and a multifocal tumor, we decided to perform ultrasound-guided radiofrequency ablation (RFA) to achieve hemostasis. The patient was admitted to the ICU for early postoperative monitoring. On the second postoperative day, the patient returned to the surgical department. In most cases, interventional treatment is necessary to achieve hemostasis, even in patients with Child-Pugh C liver function. While transarterial chemoembolization followed by staged hepatectomy is considered the treatment of choice based on current clinical evidence, RFA is a viable alternative. In this case report, we demonstrate that RFA is a safe and effective technique for achieving hemostasis. It should be considered as an option for selected patients with ruptured HCC who are hemodynamically unstable, when embolization or resection is unavailable or unfeasible due to the patient’s condition, or in cases of end-stage liver disease.

## Introduction

Hepatocellular carcinoma (HCC) is the sixth most commonly diagnosed cancer globally and the fourth leading cause of cancer-related deaths worldwide. It represents 3% of all cancers diagnosed both in Italy and worldwide, although its incidence varies by country, with a notably higher prevalence in Asian nations, where it can reach 26% of all cancers diagnosed [1]. Spontaneous liver bleeding is a life-threatening complication of HCC, occurring most frequently in adenomas and secondarily in HCCs [2]. In fact, rupture can be the first manifestation of HCC, with an incidence of 3-15% and an in-hospital mortality rate ranging from 7% to 75% [1,2], despite recent advancements in management.

The primary etiopathogenetic factors leading to HCC rupture include tumor necrosis, rapid tumor growth, vascular erosion, and occlusion of hepatic veins [1,3]. The optimal management of ruptured HCC remains a subject of debate. However, it is generally accepted that treatment should be multidisciplinary and involve two phases: (1) bleeding control and (2) specific tumor treatment after the acute phase. Procedures include emergency liver resection, transarterial chemoembolization (TACE), packing, and liver transplantation, depending on the patient’s hemodynamic stability and liver function. Radiofrequency ablation (RFA) has been described as a therapeutic alternative in selected cases, though it is typically reported in rare clinical instances or small case series [2-4].

This study aims to describe the case of a spontaneous rupture of HCC in a hemodynamically unstable patient with hemoperitoneum, which was treated with intraoperative RFA.

## Case presentation

A 74-year-old man presented to our emergency department with worsening abdominal pain that began in the morning, accompanied by a lipothymic episode (a sensation of imminent loss of consciousness and weakness). The patient reported no significant medical history or use of medications.

Upon arrival, his vitals were unstable: the patient was hypotensive (blood pressure 70/50 mmHg), tachycardic (110 bpm/min), and exhibited cold sweating, although he remained oriented to time and place (Glasgow Coma Scale 15). Physical examination revealed a globular, tense, and diffusely painful abdomen.

Arterial blood gas analysis showed anemia (hemoglobin 7 g/dl), severe metabolic acidosis, and lactates >20 mmol/L. A fast ultrasound demonstrated diffuse endoperitoneal effusion. Blood tests revealed increased creatinine, glycemia, cholestasis indices, and transaminases (Table 1). The patient was immediately given a red blood cell transfusion.

**Table 1:** Blood test results

Parameter	Results	Reference values	Items	Results	Reference values
Hemoglobin	7 g/dl	14-18 g/dl	Alkaline phosphatase	204 U/L	40-150 U/L
Creatinine	2.9 mg/dl	0.8-1.2 mg/dl	Gamma-glutamyl transpeptidase	520 U/L	8-45 U/L
Glycemia	313 mg/dl	70-99 mg/dl	Glutamic oxaloacetic transaminase	214 U/L	10-50 U/L
Total bilirubin	1.86 mg/dl	0.2-1 mg/dl	Lactate dehydrogenase	434 U/L	135-225 U/L
INR	1.36				

**Figure 1:**
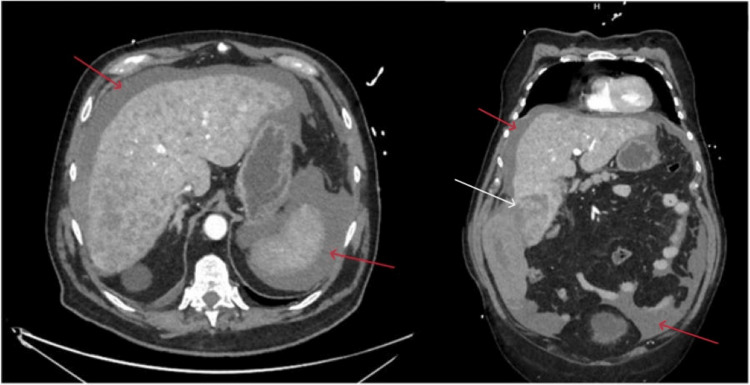
Abdominal CT scan showing hemoperitoneum (red arrows) and evidence of contrast leakage near segment 6 (white arrow)

After stabilization, a contrast-enhanced CT scan with intravenous contrast medium was performed (Figure 1). 

Enhanced CT scan revealed chronic sclerogenic liver disease with multiple confluent hypodense lesions in the liver, the largest located in segment 6. A large hematoma was noted along the right paracolic gutter, along with free abdominal effusion, which was compatible with hemoperitoneum on ascites. These findings suggested bleeding from the hepatic lesion in segment 6.

Incidental findings on the CT scan included liver cirrhosis, likely of alcoholic or metabolic etiology (Child-Pugh B - MELD 21), varices in the distal third of the esophagus (F2 with red marks), and ascites. Based on these findings, the patient was diagnosed with spontaneous rupture of HCC. Due to his hemodynamic instability and the unavailability of interventional radiology, emergency surgery was deemed necessary.

A J-shaped laparotomy was performed. Upon opening the abdominal cavity, we found hemoperitoneum, with approximately 2,800 cc of blood aspirated. The liver was cirrhotic with completely disrupted architecture. A hepatic laceration, approximately 2 cm in diameter, was located at the site of the cancerous lesion in segment 6. The hepatoduodenal ligament was identified and encircled for the Pringle maneuver. The right liver was mobilized, and hemostasis was attempted using conventional techniques (bipolar forceps, reabsorbable hemostatic agents, and fibrin glue) but was unsuccessful.

**Figure 2:**
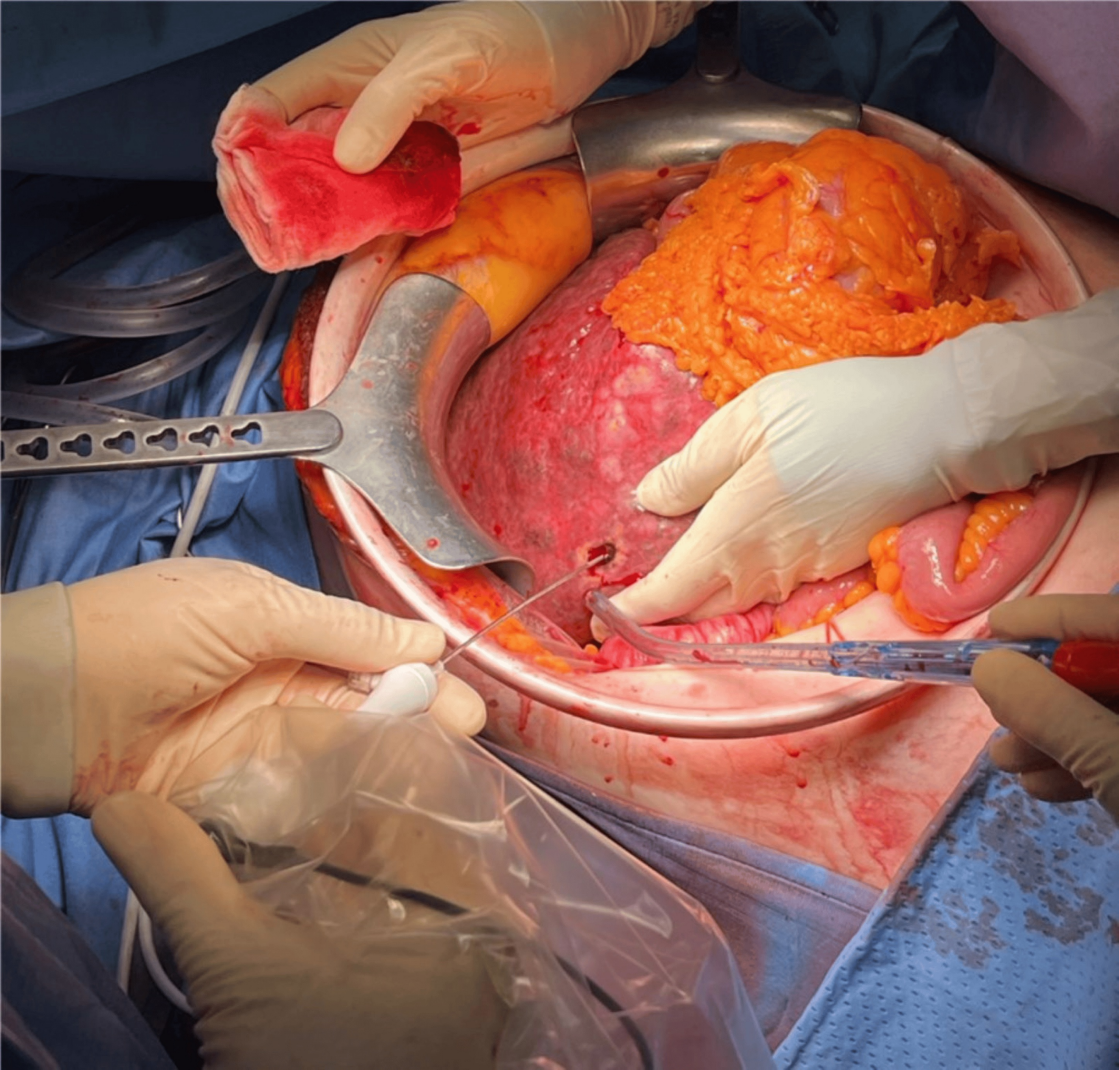
Control of bleeding from ruptured HCC using RFA with a single probe HCC: hepatocellular carcinoma; RFA: radiofrequency ablation

Due to the patient’s unstable condition, poor liver function reserve, and multifocal tumor, ultrasound-guided RFA was decided upon (Figure 2). Multiple cycles of RFA were performed using a LeVeen hook electrode needle with 4 cm exposed tips, for a total of 30 minutes, resulting in successful hemostasis. A multi-hole drain was placed, and the operation time was 140 minutes with blood loss exceeding 3,000 cc. The patient received six units of packed red blood cells and one unit of packed fresh frozen plasma.

The patient was admitted to the ICU for close postoperative monitoring. On the second postoperative day, he was transferred back to the surgical department. On the ninth postoperative day, melaena was observed, likely due to bleeding from esophageal varices, and the patient underwent esophagogastroduodenoscopy. In the subsequent days, the abdominal drainage produced ascitic, non-bloody material, and it was removed. On the 12th postoperative day, an abdominal ultrasound revealed no signs of ongoing bleeding. At the 30-day follow-up, the patient was alive, with no signs of further bleeding, although complaints related to the advanced cirrhosis persisted.

## Discussion

Ruptured HCC is a common initial presentation of the disease, occurring in 3-15% of cases. It is a life-threatening complication, associated with an overall mortality rate of 23.5%, which drops to less than 1% in patients undergoing surgical management [1,2]. All patients with spontaneous rupture are classified as T4 according to the TNM classification, irrespective of tumor size or vascular invasion, indicating a more advanced tumor stage and a poor prognosis [5]. Additionally, rupture is more common in patients with poor liver function and a high Child-Pugh score, which complicates treatment [6].

The exact mechanism behind spontaneous rupture of HCC remains incompletely understood, though several hypotheses have been proposed. One possibility is the “small room” hypothesis, which suggests that some liver segments, such as segment 6, the left lobe, and the caudate lobe, are more confined, with limited space externally bounded by the liver capsule. As the tumor grows, it increases internal pressure, causing distension of the surrounding liver parenchyma and eventual rupture of the capsule. Studies have shown that tumors larger than 5 cm are at higher risk for rupture. However, location also plays a significant role: HCCs located near the Glissonian capsule are more likely to rupture, regardless of size, while more centrally located tumors have a lower rupture risk [1].

Another hypothesis is related to vascular injury. HCCs express collagenase, which promotes the proliferation of elastin and the degradation of type IV collagen, leading to vascular damage, particularly in small arteries. This makes the vessels fragile and more prone to rupture from minor trauma or vascular stress, especially in patients with portal hypertension and cirrhosis [1]. The third hypothesis, the venous congestion hypothesis, suggests that invasion and occlusion of hepatic veins lead to venous congestion, which raises pressure within the tumor. This, in combination with tumor necrosis and coagulopathy, can result in intratumoral hemorrhage and subsequent rupture [1,3]. Given these factors, the pathophysiology of HCC rupture is likely multifactorial, combining elements from all of these hypotheses. Other contributing factors include hypertension, portal vein thrombosis, and extrahepatic invasion. Interestingly, TACE, a common treatment for HCC, has been associated with rupture due to acute ischemic necrosis and vascular injury, occurring in 0.4-0.9% of cases [1]. Additionally, according to Battula et al. [7], anticoagulant or antiplatelet therapy does not seem to significantly increase the risk of bleeding or worsen the prognosis.

Patients with ruptured HCC typically present with abdominal pain (66-100%) and abdominal distension (33%). Hemorrhagic shock may develop in 33-90% of cases, and liver failure occurs in 12-42% during the acute phase [1,3,8]. A rare but notable presentation is haemobilia, where rupture occurs within the biliary tree, resulting in Quinche’s triad: jaundice, abdominal pain, and melena. Many patients with ruptured HCC exhibit signs of liver dysfunction upon admission, which requires urgent evaluation of liver, kidney, and coagulation function [8].

The management of spontaneous ruptured HCC remains challenging and lacks definitive treatment guidelines, even in major international protocols [9-11]. The approach is still debated, and patients often present with poor clinical conditions. Treatment aims to reduce short-term mortality and improve long-term prognosis. The first step is diagnosis. If the patient’s condition permits, abdominal imaging should be performed, ideally using a contrast-enhanced CT scan to assess for active bleeding and hemoperitoneum. Ultrasound is also routinely used for the evaluation of ruptured HCC, even in patients with hemodynamic instability, and is part of the Focused Assessment with Sonography in Trauma (FAST) protocol. It can help detect intraperitoneal fluid, which may suggest hemorrhage [8]. Once diagnosed, the next step is to control the bleeding and stabilize the patient hemodynamically. Tumor treatment can often be delayed if the patient's condition does not permit immediate intervention.

**Figure 3:**
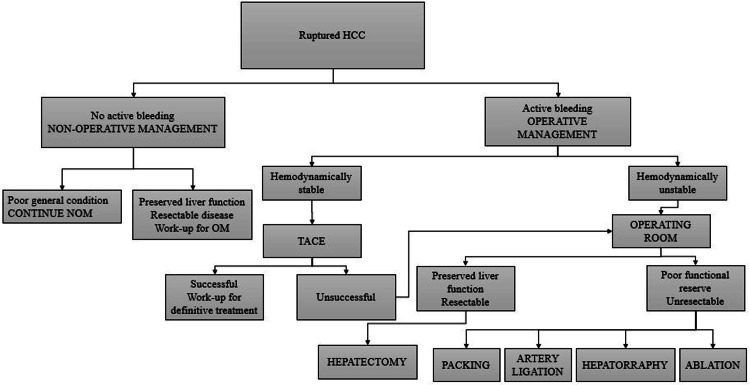
Treatment algorithm for ruptured HCC HCC: hepatocellular carcinoma; NOM: nonoperative management; OM: operative management; TACE: transarterial chemoembolization

Before the advent of modern imaging techniques, emergency surgery was the standard treatment. However, with advances in CT angiography and interventional radiology, less invasive options are now preferred. Treatment decisions should consider liver function, tumor invasion, and the patient’s overall prognosis. A management algorithm for spontaneous ruptured HCC is shown in Figure 3 [1,4,12].

If the patient is hemodynamically stable and there is no active bleeding, nonoperative management (NOM) can be considered. This approach involves volume resuscitation, blood transfusion, and correction of coagulopathy, along with the use of anti-inflammatory drugs, hemostatics, and nutritional support [8]. However, conservative treatment is associated with a high rate of re-bleeding (65.6%) and liver failure (28.1%). Additionally, hemoperitoneum increases the risk of abdominal infection and provides a pathway for the dissemination of cancer cells, raising the likelihood of abdominal implantation and metastasis. Therefore, NOM is generally reserved for patients with poor life expectancy, poor liver function, and advanced tumor stage due to its high mortality rate (85-100%) during hospitalization and a median survival of only 13 days [1].

For operative management, the first priority is to control hemostasis. Current evidence supports TACE followed by elective surgery as the gold standard for hemodynamically stable patients with ruptured HCC [3,8]. However, the ischemic effects of embolization in cirrhotic livers may predispose patients to liver failure, contributing to 20-30% of in-hospital mortality [4]. In cases of hemodynamic instability or if TACE is unavailable, emergency surgery should be considered. However, it should be noted that many patients with ruptured HCC are cirrhotic with poor functional reserve and may not tolerate surgery or may have unresectable disease. Surgical treatment is generally not recommended for patients with Child-Pugh class B disease due to poor prognosis. Hepatectomy should only be considered after achieving hemostasis, stabilizing the patient, and evaluating liver reserve. To achieve rapid surgical hemostasis, liver packing is often preferred over resection, and hepatic pedicle clamping may be necessary. A second look is typically scheduled 24-72 hours later to remove the packing and confirm local hemostasis. Other alternatives include direct suturing of the bleeding site or selective ligation of the hepatic artery [2,4].

For unstable patients with poor functional reserve, ablation therapy may offer a viable alternative. Ablative techniques, including RFA, cryoablation, microwave ablation, and irreversible electroporation, can be used. RFA is the most commonly employed method, utilizing pulsed radiofrequency current to rapidly heat tissue. At temperatures above 60 °C, proteins denature and coagulate, allowing for effective hemostasis [13]. A single probe can typically ablate approximately 3 cm of tissue, while a cluster probe is effective for lesions up to 5 cm in diameter [4]. Ablation can be performed via laparotomy, laparoscopy, or percutaneous approach, although the latter is not recommended in the management of ruptured HCC due to the risk of failure [2,4].

Several studies have explored the use of RFA for treating ruptured HCC [2,4,14-20], as summarized in Table 2.

**Table 2:** Review of the literature on RFA for ruptured HCC HCC: hepatocellular carcinoma; NA: not available; RFA: radiofrequency ablation

Study	Study type	Number of patients	Intervention	Successful hemostasis	30-day survival	One-year survival
Bertacco et al. (2017) [2]	Case report	1	RFA	100%	NA	NA
Cheung et al. (2014) [4]	Case series	19	RFA	100%	NA	NA
Ng et al. (2003) [14]	Case report	1	RFA	100%	NA	NA
Fuchizaki et al. (2004) [15]	Case report	1	RFA	100%	100%	100%
Manikam et al. (2009) [16]	Case report	2	RFA percutaneous	100%	NA	NA
Sun et al. (2009) [17]	Case report	1	RFA	100%	100%	100%
Gao et al. (2016) [18]	Case series	10	RFA videolaparoscopic	100%	100%	90%
Huang et al. (2017) [19]	Case report	1	RFA	100%	100%	100%
Kwak et al. (2019) [20]	Case series	9	RFA	66.70%	100%	83.30%

Among the studies in the literature on this topic, the majority are case reports in which RFA has been used as a rescue procedure for spontaneous ruptured HCC [2,14,15,19]. Manikam et al. reported two cases of ruptured HCC in which hemostasis was achieved using percutaneous RFA [16]. Additionally, a case of a giant ruptured HCC (with a maximum diameter of 14 cm) was treated with RFA as both a salvage and curative treatment. The procedure was repeated percutaneously over the following months to achieve ablation of the entire tumor mass. At a 56-month follow-up, the patient was healthy, demonstrating that even a giant ruptured HCC can be treated with RFA [17].

Gao et al. [18] reported a series of 10 patients who underwent laparoscopic RFA for hemorrhagic ruptured HCC. In six of these patients, the procedure successfully controlled bleeding, while in the other four, multiple sessions were needed to achieve complete ablation. Long-term results showed a three-year survival rate of 70%.

Kwak et al. [20] treated nine patients with spontaneous ruptured HCC over six years, six of whom were treated with RFA and achieved hemostasis. The other three patients had tumors greater than 10 cm in diameter and required liver resection after unsuccessful attempts to control bleeding with RFA. In their analysis, the authors noted that the RFA group had shorter operation times and required fewer blood transfusions than the surgical group. In terms of long-term survival, although both groups had a 0% recurrence-free survival rate at five years, the overall survival rate for the RFA group was significantly higher than for the hepatectomy group (83.3% vs 0%).

To our knowledge, the study by Cheung et al. [4] includes the largest series of patients. This study demonstrated improved overall hospital survival in patients who underwent RFA and required open hemostasis. Moreover, none of the patients in this group developed liver failure as a result of hemostasis with RFA. Based on these findings, the authors suggested that RFA should be considered the first-line surgical approach for patients with ruptured HCC requiring open hemostasis.

The literature indicates that RFA is a simple, safe, and minimally invasive technique that effectively achieves hemostasis in a short time. These characteristics make it suitable for patients in poor clinical condition, those with poor hepatic reserve, or those who are hemodynamically unstable [1,2]. Another advantage of RFA is its potential to treat the tumor with curative intent. In cases of small HCCs, RFA can offer oncological benefits by achieving hemostasis during tumor ablation and inactivating some cancer cells, thereby reducing the risk of tumor spread [8].

However, there are some limitations, particularly for patients with cirrhosis or large HCCs. Continuous ablation for 36 minutes has been shown to cause intolerance in cirrhotic patients, so RFA is not recommended for extended periods in these individuals. Moreover, RFA often fails to achieve effective hemostasis in giant ruptured HCCs, potentially due to the “heat sink” effect. Microwave ablation is less affected by this phenomenon and may be a better option for large ruptured HCCs [3]. In cases where hemostasis is not achieved during the first 12-minute cycle, the Pringle maneuver can be performed to reduce blood flow. If this is still insufficient, surgical treatment is recommended [4].

The use of RFA in patients with ruptured HCC has garnered increasing interest in recent years due to its efficacy and safety. However, due to the limited number of studies available in the literature, its full benefits and indications are not yet fully understood.

## Conclusions

The management of ruptured HCC remains a topic of ongoing debate. In most cases, interventional treatment is necessary to achieve hemostasis, even in patients with Child-Pugh C cirrhosis. While TACE followed by staged hepatectomy is considered the treatment of choice according to current clinical evidence, RFA presents a valuable alternative.

In our case report, we demonstrate that RFA is a safe and effective technique for achieving hemostasis. It should be considered as an option for selected patients with ruptured HCC who are hemodynamically unstable, when embolization or resection is unavailable or unfeasible due to the patient’s condition, and in those with end-stage liver disease.
